# Glances at the Hospitals

**Published:** 1905-01-07

**Authors:** 


### GLANCES AT THE HOSPITALS.

### THE LOWESTOFT HOSPITAL.

THE receipts amounted to £1,851, and the expenditure to £2,081. There was an adverse balance of about £230 on the year's working, and the committee urge upon the Governors and friends of the institution to assist the funds in every possible way. Among the receipts for the year we notice £175 from the fishing industry, and Hospital Saturday produced £80; but unless the workmen's own contributions are included in the ordinary subscriptions they do not appear at all. At the beginning of the year there were 29 patients in the hospital, and 269 were admitted, making a total of 298 under treatment. Of these 232 were discharged either cured or relieved, 5 were discharged as incurable, 30 died, and 31 remained in the wards. The report comes to us destitute of any medical or surgical statistics, and we do not see any statement relative to the cost per patient or of the average duration of treatment.

### WAKEFIELD AND CLAYTON HOSPITAL.

THE total receipts, exclusive of legacies, were £5,181, and the total expenditure was £4,648, being £320 in excess of last year. The average cost per bed occupied was £70 4s., and the average cost per in-patient was £5 18s. 8d. The estimated cost of each out-patient was 3s. 10d. At the beginning of the year the hospital contained 50 patients, and 626 were admitted during the year, making a total of 676 under treatment. Of these 441 were discharged recovered, 108 relieved, 18 not relieved, 50 remained in the wards and 59 died. Of these 16 died within 48 hours of admission. There were 6 cases of rheumatic fever, all being cured; 13 cases of pneumonia and broncho-pneumonia, of whom 9 died; 9 cases of strangulated hernia, of whom 3 died. These examples will show that the medical and surgical tables give results of treatment, but the operation table tells us nothing beyond the numbers operated on for the various diseases or accidents named. Altogether there were 169 operations on the in-patients, and there is no anæsthetic table.

